# Fanning the Flames of Passion: A Develop Mindset Predicts Strategy-Use Intentions to Cultivate Passion

**DOI:** 10.3389/fpsyg.2021.634903

**Published:** 2021-07-14

**Authors:** Patricia Chen, Yuching Lin, Don J. H. Pereira, Paul A. O’Keefe, J. Frank Yates

**Affiliations:** ^1^Department of Psychology, National University of Singapore, Singapore, Singapore; ^2^Institute for Applied Learning Sciences and Educational Technology, National University of Singapore, Singapore, Singapore; ^3^Department of Psychology, University of Virginia, Charlottesville, VA, United States; ^4^Division of Social Sciences, Yale-NUS College, Singapore, Singapore; ^5^Department of Management and Organisation, NUS Business School, National University of Singapore, Singapore, Singapore; ^6^Department of Psychology, University of Michigan, Ann Arbor, MI, United States

**Keywords:** mindsets, implicit theories, passion, self-regulation, strategies, well-being

## Abstract

College students are encouraged to major in subjects they are passionate about but less often advised about what to do when passion is low. What self-regulatory strategies do students use to up-regulate their passion toward their subjects, and how might they be oriented toward using such effective strategies? Three studies examined how the belief that passion is developed – a “develop” mindset – relates to students’ intentions to use strategies to actively grow their passion. The more strongly students endorsed a develop mindset, the more of these “cultivation strategies” they reported using, and in turn, the larger their increase in reported passion toward their subject majors (Study 1). Instilling a develop mindset causally increased students’ intentions to use more cultivation strategies (Study 2) – with some effects lasting up to a year (Study 3). Instilling a develop mindset can potentially help students to ignite their passion when its flame burns low.

## Introduction

College students today are bombarded with advice to chase their passions. They are constantly urged by successful industry role models, graduation speakers, and the popular media to major in subjects that they are passionate about (e.g., Wragg and Provenzano, 2014; Siena College, 2016), and to vigorously pursue careers in their passions (Jobs, 2005; Pausch, 2007). For example, the late Apple co-founder and CEO, Steve Jobs, recommended, “… the only way to do great work is to love what you do. If you haven’t found it yet, keep looking.” Such well-intended advice can carry the implicit assumption that once passion is “found,” it remains consistently high (O’Keefe et al., 2018). But passion, like any other motivational state, can wax and wane over time. During periods of low passion, what can students do to regulate their passion?

How students respond when their passion is low may depend on their beliefs (or “mindsets”) about passion. If they do not believe that passion can be developed, they may be less motivated to cultivate it. But if they believe passion can be developed, they might be more motivated to regulate it through the use of effective strategies – such as thinking about how a subject might be relevant to their personal goals or seeking out inspirational mentors and peers. The more students use effective strategies to cultivate their passion, the more we would expect their passion to increase over time. Our studies investigated how students’ belief that passion can be developed – a “develop mindset” – can be related to their reported use of various strategies to actively (rather than passively) and deliberately cultivate passion, especially in moments when passion is low.

## The Experience of Passion

Passion is the experience of strongly identifying with an activity, feeling motivated to engage in it, and experiencing positive affect when doing it (Chen et al., 2020; Vallerand et al., 2003). In the case of students, this involves a strong identification with a particular subject, high levels of motivation to learn it, and positive affect when learning. The experience of passion goes beyond interest because it involves a deeper personal identification with the subject, and passion often engages more intense affect (Vallerand, 2017). Importantly, passion is associated with a host of benefits – including greater persistence, commitment, positive affect, the experience of flow, well-being, physical health, and performance (Vallerand and Houlfort, 2003; Cardon et al., 2009; Perttula and Cardon, 2011; Vallerand et al., 2014; Chen et al., 2015; Vallerand, 2017). Hence, passion has become a highly valued experience, and it is even considered an “ingredient for success” by some in society today (Jobs, 2005; Schwantes, 2019).

However, as is the case with many motivational states, the intensity of one’s passion may not always remain stable or high (Meece et al., 1990; Pokay and Blumenfeld, 1990; Wolters, 1998). When students lack passion toward a subject, they may not be as engaged in learning (Stoeber et al., 2011), show lower levels of persistence (Cardon and Kirk, 2015), or even question their identity (if they had previously identified with the subject; Vallerand et al., 2003; Vallerand, 2010). These moments of low passion can potentially be crucial turning points when a student might decide to disengage in learning or give up entirely on majoring in a subject toward a profession. Yet, such low-passion experiences are much less often talked about, let alone addressed, in popular media or scientific research.

When passion is low, what do people do – and what can they do – to help themselves cultivate more passion? Can we change the way people think about passion to help them use effective strategies to regulate it when it is low? In our research, we ask, and provide some initial answers, to these empirical questions. In doing so, our research contributes to the science on passion, which to our knowledge, has not focused on understanding how people actively cultivate their passion when it is lacking.

## Strategies to Cultivate Passion: Lessons From Interest Regulation

How can people increase their passion? The literature on how passion is cultivated has generally focused on the role of the social environment (e.g., parental autonomy support: Mageau et al., 2009; Vallerand, 2017), personality (e.g., perfectionistic tendencies and signature strengths; Forest et al., 2012; Verner-Filion and Vallerand, 2016), self-efficacy beliefs and perceptions of competence (Thorgren and Wincent, 2013; Dalborg and Wincent, 2014; Cardon and Kirk, 2015), and task characteristics (e.g., autonomy to perform the task and task demands; Fernet et al., 2014; Treìpanier et al., 2014). While these approaches are valuable, many tend to focus on less controllable factors (such as external task characteristics or parental nurturance) or self- and other-perceptions based on past experiences and successes. They do not focus on addressing the strategies that individuals use to purposefully grow their passion – such as seeking out opportunities, selecting their social interactions or environments, changing their attitudes, and more.

In this research, we investigated what kinds of strategies people use to regulate their own levels of passion – specifically to increase (or “up-regulate”) passion. Our research contributes an understanding of the student as an active self-regulator of passion. We identify strategies that students autonomously employ to increase their passion toward their subjects, along with their mindset about passion (defined as a frame of mind that orients a person toward a particular set of associations and expectations; Molden and Dweck, 2006; Crum et al., 2013) that sets the stage for such adaptive strategies.

Given the dearth of research on concrete ways in which people regulate passion, we (a) borrowed ideas from the rich theory on interest development to understand possible processes by which students might also increase their passion toward their subjects; and we (b) complemented this with an open-ended exploration in our first study about the kinds of strategies that students reported using. Although passion is not the same as interest, some of the ways in which students increase passion toward their subjects may be similar to the kinds of strategies that they use to actively increase their interest in an activity. For example, one of the most critical, well-studied factors is their appreciation for how the subject relates to their life and personal goals (Hulleman and Harackiewicz, 2009; Hulleman et al., 2010) or to society (Yeager et al., 2014a). They might also gain familiarity with the subject (Zajonc, 1968; Reber and Lewis, 1977), or seek out inspirational teachers or motivating environments (e.g., Midgley et al., 1989; Blumenfeld et al., 2005), for example. Granted, there may also be other strategies for cultivating passion that differ from those effective for cultivating interest. Therefore, it was important that we complemented theoretical ideas from the interest development literature (see Hidi and Renninger, 2006; Renninger and Hidi, 2015) with an analysis of students’ open-ended descriptions of their natural strategy-use when regulating passion.

As mentioned earlier, the kinds of strategies that students generate and apply when their passion is low may depend on whether they believe that passion can actually change or not. If they do not believe that passion is developed over time, then they may be less inclined to try any strategies to cultivate their passion. But if they believe that passion is developed, we predict that they would be more likely to think of and to apply strategies to actively up-regulate it when it is low (e.g., focusing on the personal relevance of the subject, building their familiarity and skill in the subject, or seeking out inspirational teachers). In addition to measuring the kinds of strategies that students are inclined to use to up-regulate passion, we also measured students’ mindset that passion is developed, which we predicted would incline them toward using such active and deliberate regulatory strategies to cultivate passion.

## Mindsets About Passion

Mindsets are people’s fundamental beliefs about themselves and the world (Furnham, 1988), which orient them toward using certain kinds of self-regulatory strategies over others during goal pursuit (Dweck and Leggett, 1988). For example, a “growth” (as compared to a “fixed”) mindset about intelligence – the belief that intellectual abilities are malleable, not set – predicts a mastery orientation (as opposed to a performance orientation), greater persistence, and positive academic outcomes (Burnette et al., 2013; Dweck and Yeager, 2019). Likewise, a “growth” belief that problems in romantic relationships can be overcome is associated with greater commitment to a partner, greater persistence through obstacles, and the use of adaptive coping strategies to overcome conflict (Knee et al., 2003). This has often been compared to an orthogonal “destiny” belief that relationship partners are either destined to be together or not (Knee, 1998). Research across multiple domains has shown that the belief that a particular attribute can develop inclines people toward using strategies to actively cultivate that attribute, especially in challenging times (e.g., Molden and Dweck, 2006; Burnette et al., 2013).

Similarly, people also have mindsets about how passion is achieved (Chen et al., 2015). A “develop” mindset about passion is the belief that passion is *developed* over time toward a career or subject major. This is contrasted with a “fit” mindset about passion, which is the belief that passion is found through the *fit* with the “right” career or subject major. These mindsets about passion have important implications for people’s expectations, choices, and well-being (Chen et al., 2015). Prior studies on mindsets of passion, which primarily studied working adults, found that working adults who endorsed a develop mindset (more than a fit mindset) forecasted that their passion toward an unenjoyable line of work would grow over time. Hence, these adults were less inclined to prioritize immediate enjoyment in a line of work, and preferred other important vocational characteristics, such as pay. In contrast, working adults who endorsed the fit mindset (over the develop mindset) expected their passion toward a job to remain high over time, hence they tended to prioritize immediate enjoyment over other vocational characteristics when choosing a job. Both the develop and fit mindsets significantly predicted people’s self-reported vocational passion, satisfaction, and commitment toward their vocations – indicating that people with either dominant mindset are able to achieve passion toward their work, just through different means (Chen and Ellsworth, 2019).

Drawing from and extending these previous theories, we predicted that a develop mindset would be particularly relevant to the goal of actively regulating passion when it is low. We hypothesized that the more strongly students believe that passion is developed, the more they would be inclined to leverage strategies aimed at actively and purposefully up-regulating their passion – what we term “cultivation strategies.” Examples of these cultivation strategies are thinking about how the subject relates to their personal goals or to improving society, building experience in and familiarity with the subject, or seeking out inspirational mentors and peers. This hypothesis is consistent with prior theories that link growth mindsets (of intelligence, personality, leadership, empathy, and more) to the mastery goal of working on and improving those skills (Dweck et al., 1995; Blackwell et al., 2007; Hoyt et al., 2012; Schumann et al., 2014; Schroder et al., 2017). Comparatively, a fit mindset might not be as relevant (and therefore unrelated) specifically to the goal of actively cultivating passion, because it tends to be associated with seeking a match with one’s interests from the outset, rather than actively growing passion over time. We are not proposing that a fit mindset lacks value – but rather pointing out that it should theoretically be less relevant specifically to the goal of actively up-regulating passion (and perhaps more useful for activating different kinds of strategies or goals that are not the focus of the current studies).

To our knowledge, this research is the first to articulate and empirically test these relationships between the develop (and fit) mindset of passion and people’s intentions to use various strategies to regulate their passion. It also generalizes prior work on mindsets about passion beyond working adults to students, who are at a key developmental period of building knowledge and skills in college toward the professional careers that they want to pursue.

## Overview

This research brings us one step closer to answering the big questions that we posed earlier: When passion is low, what *do* people do – and what *can* they do – to regulate their passion? Can we change the way people think about passion to help them better regulate it when it is low? Our studies (1) investigated the various self-regulatory strategies that students report using to regulate their passion, and (2) specifically tested the prediction that a develop mindset about passion is associated with students’ intended and reported use of cultivation strategies. In correlational Study 1, we surveyed a sample of college students to understand (1) the kinds of cultivation strategies that they naturally report using to regulate their passion when it is low, and (2) how a develop mindset naturally relates to these cultivation strategies. In experimental Studies 2 and 3, we tested whether instilling a develop mindset could causally increase students’ intentions to use cultivation strategies, relative to a fit mindset, in the short-term and 1 year later, respectively.

## Study 1: A Develop Mindset and Cultivation Strategy Use

The first naturalistic, correlational study had two goals: One, to understand the various cultivation strategies that students report naturally using to up-regulate their passion toward their subject majors. Two, to test our hypothesis that a develop mindset is associated with how much students report using cultivation strategies in general, which in turn, should be associated with their reported increases in passion toward their subjects.

### Participants

We recruited 316 undergraduates from seven academic units (Engineering, Business, Natural Science, Social Science, Humanities, Nursing, and Undeclared Majors) at a large U.S. Midwestern public university to complete our online survey. We restricted the survey to third- and fourth-year students, who tend to have more experience with their college majors and therefore more time to form attitudes toward these subjects. Data from seven Year 2 students who participated (despite not being eligible) were excluded leaving 309 upper-class students for analyses (gender: 27.5% male, 52.8% female, 1.0% other, and 18.8% missing; *M_*age*_* = 21.0, age range: 19–30 years).

### Procedure

As part of a larger survey, we measured students’ (develop and fit) mindsets about passion, the strategies they had employed to regulate their passion, and reported changes in their passion toward their college majors from the time they had started in their majors to the time they took the survey.

### Measures

#### Mindsets About Achieving Passion

To assess students’ mindsets about passion toward their subjects, we adapted the single-item mindset measure from an established measure for passion for work (Chen et al., 2015). In previous research, these single-item measures were validated against multi-item mindset scales and a dichotomous forced-choice measure (Chen et al., 2015). We measured endorsement of the develop and fit mindsets, respectively, with the questions: “Passion for a subject is something that you must develop” and “A subject that you are naturally passionate about exists. You must simply find it.” Both measures were answered on 1 (*Strongly disagree*) to 6 (*Strongly agree*) scales. In later studies in this paper, we expanded on these single-item measures to create multi-item mindset scales to assess students’ beliefs about passion toward their subjects.

#### Changes in Passion

Students were asked to report their current or intended college major. Out of those who answered the question, 89.8% listed a current major and 10.2% listed an intended major. Students rated how passionate they felt toward this major when they first started learning it (“initial passion”), and how passionate they felt at the time of the survey (“current passion”), using 0 (*Not at all*) to 6 (*Extremely*) scales. To reduce possible demand effects, these ratings were placed in a different section of the survey, separate from the mindset scales. We computed a passion change score for each participant by subtracting initial passion from current passion. According to Huck and McLean (1975), change scores provide the same useful information as a repeated-measures ANOVA in a “more straightforward, parsimonious manner” (p. 517). An issue with the use of change scores is its possible reduced reliability when compared to the two constituent measures themselves (Cattell, 1982; Grigorenko and O’Keefe, 2004; Thomas and Zumbo, 2012). However, in certain instances, change scores can be validly used – such as in the case of sufficiently powered studies (Thomas and Zumbo, 2012). As Study 1 meets these criteria, the results of our change score analysis can be considered valid.

#### Strategies for Increasing Passion

Students responded to an open-ended question about the reasons for their change in passion (or lack thereof): “What do you think is responsible for the change or lack of change in your passion?” In order to avoid demand effects of directly asking students what they had done to regulate their passion, we left our question open-ended and framed it in a way that allowed students to describe any actions they took, along with other possible reasons (e.g., events out of their control).

Because we were interested in how students cultivate passion, we focused on coding the responses of the 61.5% of students who reported an increase in their passion. Two independent coders coded these responses into seven categories of strategies that emerged in a bottom-up fashion from thematic analyses: (a) recognizing personal relevance in learning the subject, (b) recognizing the societal relevance of the subject, (c) building familiarity with the subject, (d) gaining practical experience in applying the subject, (e) seeking or recognizing the influence of teachers or environments, (f) focusing on parts of the subject that they naturally like, and (g) performing well in the subject (1 = theme present in response or 0 = theme not present in response; κs ranged from 0.73 to 0.92). Table 1 shows how frequently each of the different themes occurred in participants’ responses. Each response could be included in more than one category. Discrepancies in coding were resolved through discussion.

**TABLE 1:** Students’ reasons for why their passion had increased over time (*N* = 128).

Reasons	Occurrence percentage (*n*)
(a) Appreciating the personal relevance of the subject	10.9 (14)
(b) Appreciating the societal relevance of the subject	14.8 (19)
(c) Building familiarity with the subject	35.2 (45)
(d) Gaining practical experience in applying the subject	10.9 (14)
(e) Seeking or recognizing teacher or environmental influence	20.3 (26)
(f) Focusing on parts of the subject that they naturally liked	7.0 (9)
(g) Performing well in the subject	7.0 (9)

*Note: *n*, number of occurrences. Responses could be coded into more than one category (such that the sum of proportions exceeds 100%): 26.6% of responses fell into two categories and 7.0% fell into three.*

An example of a student’s response that was coded as recognizing the personal relevance of the subject was: “I was able to see how the material I was using could be applied to my current job and my future professional aspirations.” An example of a student’s response that was coded as recognizing the societal relevance of the subject was: “I knew I was interested in the topic and the more I learned, the more I appreciated the subject in terms of real-world impact.” An example of a student’s response that was coded as building familiarity with the subject was: “The more I learned about it, the more I realized how much I enjoyed it.” An example of a student’s response that was coded as gaining practical experience in applying the subject was: “my experience at my internship helped to grow my passion in mechanical engineering.” And an example of a student’s response that was coded as seeking or recognizing the influence of teachers or environments was: “I have some great friends that are very passionate about the topic and they inspire me to learn more about it.” More examples of responses from each category are described in the Supplementary Appendix A. Many of these themes about how students reported regulating their passion overlapped with scientific findings on effective strategies for regulating interest – such as connecting the subject content to personal goals or to societal impact (Hulleman and Harackiewicz, 2009; Hulleman et al., 2010), gaining familiarity with the subject content (Zajonc, 1968; Reber and Lewis, 1977), and seeking out people who can inspire you (Midgley et al., 1989).

Of the seven coded strategies, we classified five themes as “cultivation” strategies (see a–e above) because these were active, deliberate strategies that students used to increase their passion over time. To compute our key outcome measure, we created an overall index of “cultivation strategy-use” for each student by summing the total number of cultivation strategies they described using (range of strategies reported: 0 – 3; *M* = 0.44, *SD* = 0.71). Categories (f) and (g) were not classified as cultivation strategies because these strategies did not involve actively increasing passion through students’ efforts beyond what they were already inclined toward. For example, simply scoring well could reflect an easy test or extrinsic motivation to perform well, even if students were not interested or passionate about the material; and a student may limit themselves to reading just the two topics that they were already passionate about, ignoring the numerous other topics covered in the textbook – areas that actually need up-regulating. These non-cultivation strategies were not the main focus of this research, hence we do not discuss them further. When we regressed students’ passion change scores on each of the five cultivation strategies, each cultivation strategy significantly related to increases in passion – validating students’ self-reported strategies to increase their passion (see Table 2).

**TABLE 2:** Study 1 linear regression results from predicting passion change scores with students’ reported use of each category of strategy in separate models.

Reasons	*b*	*SE*	95% CIs of *b*
(a) Appreciating the personal relevance of the subject	2.25***	0.44	[1.38, 3.12]
(b) Appreciating the societal relevance of the subject	1.97***	0.36	[1.27, 2.67]
(c) Building familiarity with the subject matter	1.30***	0.24	[0.83, 1.78]
(d) Gaining practical experience in applying the subject	1.45**	0.43	[0.61, 2.29]
(e) Teacher influence or educational environment	1.27***	0.33	[0.62, 1.91]

Note: *b*, unstandardized beta coefficient; *SE*, standard error of *b*. **p < 0.01. ***p < 0.001. Bonferroni corrections for multiple tests showed the same results (all *p*s < 0.01).

## Results

Our results were the same whether we included all students (those who reported a current or intended major) or only those who reported a current (but not intended) major. Hence, we included all students in the analyses reported here. Consistent with previous research (Chen et al., 2015), students’ responses on the develop and fit mindset single-item measures were uncorrelated, *r* = 0.00, *p* = 0.951. On average, students endorsed the fit mindset (*M* = 4.70, *SD* = 1.04) more strongly than the develop mindset (*M* = 4.22, *SD* = 1.02), *M_*diff*_* = 0.48, 95% CI [0.31, 0.64], *SD_*diff*_* = 1.46, paired *t*-test *t*(298) = 5.67, *p* < 0.001, *d* = 0.33. This is consistent with prior findings showing that American adults tend to endorse the fit mindset more strongly than the develop mindset, on average (Chen et al., 2015).

We tested our primary hypothesis by regressing participants’ cultivation strategy-use index on their develop mindset scores. As predicted, students with a stronger develop mindset reported using more cultivation strategies in general, *b* = 0.16, [0.09, 0.24], *SE* = 0.04, *t*(298) = 4.14, *p* < 0.001. A one-unit increase in the endorsement of the develop mindset was related to listing 0.16 more cultivation strategies that they had tried or experienced. However, a fit mindset did not predict students’ use of more cultivation strategies, *p* = 0.522, which we also expected.

We present additional exploratory analyses testing the relation between a develop mindset and students’ reported use of each cultivation strategy in Supplementary Appendix B. The pattern of results indicates that the more students endorsed a develop mindset, the more likely they were to report using each of these specific cultivation strategies, though the sizes of these relations differed by strategy. For example, the more students endorsed a develop mindset, the more likely they were to describe appreciating the societal relevance of the subject (*b* = 0.63, *SE* = 0.27, Wald (1) = 5.55, *p* = 0.018, odds ratio = 1.87, 95% CI for odds ratio: [1.11, 3.15]), and gaining practical experience in the subject (*b* = 1.09, *SE* = 0.36, Wald (1) = 9.31, *p* = 0.002, odds ratio = 2.97, [1.48, 5.97]). However, a fit mindset did not predict students’ use of individual cultivation strategies, *ps* > 0.160.

As predicted, endorsement of a develop mindset was positively associated with changes in passion, *b* = 0.20, [0.01, 0.40], *SE* = 0.10, *t*(259) = 2.03, *p* = 0.044; but endorsement of a fit mindset was unrelated to it, *b* = 0.16, [−0.04, 0.35], *SE* = 0.10, *t*(258) = 1.57, *p* = 0.119.

**FIGURE 1:**
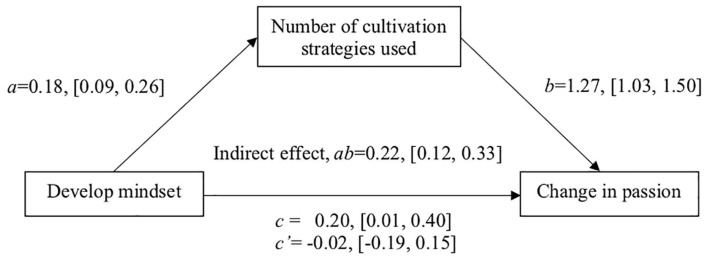
Unstandardized regression coefficients and their 95% confidence intervals representing the effect of a develop mindset on students’ change in passion scores over time, mediated by the number of cultivation strategies students described using.

We conducted indirect effects analyses to test the hypothesis that a develop mindset is associated with how much students report using cultivation strategies, which in turn, should be associated with reported increases in passion over time. As described above, students with a stronger develop mindset reported using more cultivation strategies. The more cultivation strategies they reported using, the greater the increase in their changes in passion, controlling for their develop mindset, *b* = 1.27, [1.03, 1.50], *SE* = 0.12, *t*(258) = 10.44, *p* < 0.001. The direct effect of the develop mindset on students’ changes in passion was non-significant after controlling for the number of cultivation strategies students reported using, *b* = −0.02, [−0.19, 0.15], *SE* = 0.09, *t*(258) = −0.24, *p* = 0.814. Indirect effects analyses using PROCESS with 10,000 bootstrap resamples (Hayes, 2013) showed that the number of cultivation strategies used significantly mediated the effect of a develop mindset on their changes in passion, bootstrapped indirect effect *ab* = 0.22, 95% CI = [0.12, 0.33] (see Figure 1).

## Discussion

First, we identified seven strategies to which students attributed their increases in passion, five of which were oriented toward actively cultivating passion. Second, as predicted, endorsing a develop mindset (but not a fit mindset) was associated with naturally using more of such cultivation strategies. In turn, the more cultivation strategies students described using, the greater their reported increases in passion over time. Students’ reported use of cultivation strategies significantly mediated the relation between a develop mindset and increases in their passion. These mediation findings should be interpreted with caution considering the limitations of correlational mediation analyses (e.g., the mediator is one out of many possible other unobserved variables that could affect these outcomes; Bullock et al., 2010). Nevertheless, these findings underscore the important role that the develop mindset naturally plays in orienting students toward using cultivation strategies for up-regulating their passion.

Also as expected, the fit mindset was not significantly related to the use of cultivation strategies. As prior research suggests, a fit mindset may be associated with different self-selection strategies (e.g., exploring other subjects) when a subject no longer elicits passion, rather than staying with the same subject and actively growing passion toward it.

For the reasons described above, we used change scores to compute changes in students’ self-reported passion over time. Future research could potentially utilize more sophisticated tools such as Structural Equation Modeling to model change scores as a latent variable (which can theoretically limit the degree of measurement error of the change scores, resulting in a highly reliable factor; McArdle, 2009; Renner et al., 2012).

Granted, these findings were correlational, so the direction of the causal relation between a develop mindset and students’ reported use of cultivation strategies remained an open empirical question, to which we turned next. Studies 2 and 3 extended these correlational, retrospective self-report findings to experimentally test the causal relation between a develop mindset and students’ inclination to use cultivation strategies.

## Study 2: Priming a Develop Mindset Increases Students’ Intentions to Use Cultivation Strategies

Study 2 tested the causal effects of priming a develop mindset (vs. fit mindset) on students’ intentions to use cultivation strategies. We hypothesized that participants who had a develop mindset primed would be more inclined toward using cultivation strategies, compared to those who had a fit mindset primed. Random assignment of participants to condition ruled out the possibility of effects driven by individual differences on our dependent measures.

### Participants

To have a sufficiently large sample, we recruited students from introductory psychology participant pools from two schools in the U.S., including a large public Midwestern university (*n* = 52), and a community college on the west coast (*n* = 118). Our sample analyzed comprised 152 participants who answered at least one question in the survey (gender: 35.5% male, 62.5% female, 1.3% other, 0.7% no response; *M*_*age*_ = 22.7 years, *SD* = 6.62 years). Supplementary Table 1 summarizes the demographic breakdown of each school. The study, including random assignment, was administered online through the computer, ensuring that experimenters were blind to participants’ condition.

### Procedure

Participants were randomly assigned to one of three conditions: Develop, Fit, or a Well-being condition (the well-being condition was not the focus of our hypothesis-testing in this research, hence we do not discuss it further). For transparency, results from exploratory analyses comparing the two mindset conditions with this “well-being only” condition are summarized in the Supplementary Table 2 (for this study) and Supplementary Table 3 (for Study 3). Here, we focused on the comparison between activating a develop mindset versus a fit mindset on students’ intentions to use cultivation strategies. Participants first read an online article corresponding to their assigned condition. Those in the Develop condition read an article emphasizing that passion develops over time, stating that “the science shows that we can learn to love our subjects or lines of work over time” and “passion toward any subject or line of work develops through a gradual process.” Those in the Fit condition read an article emphasizing that passion comes from finding a field that is the right fit for oneself, for example: “The science shows that we can find our passion – it’s mostly about listening to our intuitions and looking out for the right fit” and “students and professionals have found their passions in careers that are compatible with their interests and personalities.” These articles were similar in length and format, and designed to look like popular psychology articles (refer to Supplementary Appendix C for the Develop and Fit condition articles); their designs were inspired by earlier successful mindset manipulations that also used persuasive articles (e.g., Bergen, 1991; Hong et al., 1999). After reading the article, students engaged in a saying-is-believing exercise by summarizing the main message of the article in their own words as advice for a younger student struggling to experience passion toward their subjects. Such saying-is-believing exercises can elicit self-persuasion effects and promote internalization of the message espoused (Wilson, 1990; Aronson, 1999).

As manipulation checks for the develop and fit article primes, participants completed our 3-item develop and fit mindset scales. These manipulation checks were adapted from and expanded upon Study 1’s single-item measures, and all items were completed on a 1 (*Strongly disagree*) to 6 (*Strongly agree*) response scale. The 3-item develop mindset scale included items such as “Passion for a subject is usually something that you must develop” and “Passion for a subject is usually developed over time;” the 3-item fit mindset scale included items such as “Passion for a subject is found through a fit with the right subject for you” and “Passion for a subject is something that you find by choosing a subject that naturally suits you as a person.”

Our main outcome variable was students’ future intentions to use cultivation strategies when their passion for their subjects was low. Participants read five vignettes, each describing one of the five cultivation strategies (a–e) identified in Study 1. In each vignette, we described each strategy in the third person to reduce demand effects and social desirability (which could be higher if we had presented each strategy from the first-person perspective). As an example, participants read the following vignette about the cultivation strategy of focusing on the personal relevance of the subject:

“Student C doesn’t feel very passionate toward a particular subject and would like to increase their passion toward that subject. This semester, Student C actively focuses on how every class that C is taking in that subject is relevant to C’s own future goals (such as C’s career goals and personal development goals).”

For each cultivation strategy, participants made three ratings about their own intentions to use the strategy on 0 (*Not at all*) to 6 (*Extremely*) response scales (e.g., “How likely are you to use Student [C]’s strategy of [*description of strategy*] when you feel little passion toward your own subjects?”). For each vignette, the three items were highly correlated (*r*s ranged from 0.61 to 0.84; all αs > 0.84), so we averaged them. As we were primarily interested in how much students were inclined to use cultivation strategies in general, we summed these strategy ratings across the five cultivation strategies to form a “cultivation strategy-use intentions” composite score per student. In additional exploratory analyses, we present the results of each cultivation strategy separately, to understand which specific strategies might be driving our effects.

## Results

### Message Content

As a first manipulation check, two independent raters coded students’ open-ended messages for content related to the develop mindset (*κ* = 0.91) and fit mindset (*κ* = 0.91). Coding discrepancies were resolved through discussion. As expected, participants in the Develop condition were much more likely to mention the develop mindset than participants in the Fit condition (Develop condition: 83.0%; Fit condition: 7.7%), χ^2^(1) = 60.01, *p* < 0.001; participants in the Fit condition were much more likely to mention the fit mindset in their messages than participants in the Develop condition (Develop: 11.3%; Fit: 84.6%), χ^2^(1) = 56.53, *p* < 0.001.

### Mindset Scales as Manipulation Checks

Mean composites were calculated for the develop mindset and fit mindset multi-item scales (develop mindset scale: α = 0.87; fit mindset scale: α = 0.79). Confirming the success of our manipulation, students in the Develop condition (*M* = 4.95, *SD* = 0.72) endorsed the develop mindset to a greater extent than those in the Fit condition (*M* = 4.03, *SD* = 1.04), *b* = 0.93 [0.58, 1.27], *t*(104) = 5.37, *p* < 0.001. These results suggest that, as expected, the develop mindset article was effective at priming a develop mindset, relative to the fit mindset article. In the Fit condition, although the mean endorsement of the fit mindset scale (*M* = 4.69; *SD* = 0.79) was higher than that in the Develop condition (*M* = 4.44; *SD* = 1.00), this difference was small and non-significant, *p* = 0.171. In other words, there were no differences in students’ endorsements of the fit mindset. It is plausible that this fit mindset article did not sufficiently make the fit mindset message salient enough; but it is also plausible that these small effects could be due to the natural prevalence of the fit mindset message among our American student samples (Chen et al., 2015). Hence, repeating such a familiar message, even in a stronger form, may not substantially shift students’ beliefs toward a stronger fit mindset. Nevertheless, these findings importantly point to the effects of the develop mindset article prime on increasing develop mindset endorsement, but not changing fit mindset endorsement.

### Cultivation Strategy-Use Intentions by Condition

As predicted, participants in the Develop condition (*M* = 27.13, *SD* = 3.57) reported on average greater intentions to use cultivation strategies in general, compared to those in the Fit condition (*M* = 24.21, *SD* = 4.12), *b* = 2.92, [1.44, 4.40], *t*(104) = 3.90, *p* < 0.001. These results provide empirical evidence for our hypothesis that activating a develop mindset can causally increase people’s intentions to use cultivation strategies.

To examine which strategies might have driven the effect, we additionally present exploratory results broken down by individual cultivation strategy in Table 3. As shown in Table 3, students in the Develop condition reported, on average, a greater likelihood of appreciating the personal relevance of the subject, building familiarity with the subject matter, seeking out inspiring teachers or environments, and gaining practical experience in applying the subject.

**TABLE 3:** Study 2 group means (Standard Deviations) and regression contrasts comparing participants’ cultivation strategy-use intentions between the Develop and Fit conditions.

Strategy	Develop condition	Fit condition	Planned contrast (*b* and 95% CI)
(a) Personal relevance	5.35 (1.02)	4.74 (1.44)	0.62 [0.14, 1.10]*
(b) Societal relevance	5.32 (1.18)	5.09 (1.13)	0.24 [−0.21, 0.69]
(c) Build familiarity	4.93 (1.37)	4.01 (1.58)	0.94 [0.36, 1.51]**
(d) Practical experience	5.72 (0.98)	5.12 (1.41)	0.63 [0.16, 1.10]*
(e) Teachers or environments	5.81 (0.87)	5.35 (1.16)	0.45 [0.05, 0.84]*

Note: Unstandardized beta coefficients are presented with their 95% confidence intervals in square brackets. *p < 0.05; **p < 0.01.

## Discussion

Instilling the develop mindset through a brief article manipulation increased students’ endorsements of a develop mindset (without changing their fit mindset), and their intentions to use cultivation strategies, relative to priming the fit mindset. These findings underscore the causal relationship between this develop mindset and students’ intentions to apply effective strategies for up-regulating passion when it is low. Nonetheless, these short-term findings also provoke another question: Can promoting a develop mindset (vs. a fit mindset) produce longer-term changes in students’ intentions to use cultivation strategies? We tested this in Study 3, where we conducted a year-long experiment, to examine the potential long-term implications of instilling a develop mindset on students’ intentions to use cultivation strategies.

## Study 3: Long-Term Effects of Priming a Develop Mindset

We used a similar study design in this longitudinal experiment as in Study 2. In Part 1, which was conducted in the Fall academic semester, we randomly assigned students to the Develop or Fit conditions for comparison (or a third Well-Being condition for different hypotheses that are not the focus of this paper), where they read and summarized an article. In Part 2, 1 year later, we asked students to complete a follow-up survey, in which they rated their intentions to use the various cultivation strategies as described in Study 2 to test the potential long-term effects of instilling a develop mindset on their strategy-use intentions. We focused on differences between the Develop and Fit conditions on their intentions to use cultivation strategies 1 year post-prime.

## Part 1: Random Assignment to Mindset Condition

### Participants

We had planned to recruit as many participants from the psychology subject pool as possible, given the course credits assigned to each researcher. We recruited a total of 240 undergraduates from a large Midwestern U.S. public university for course credit. Excluding two participants who completed the survey twice and had been exposed to more than one experimental condition, 238 participants remained for analyses (gender: 54.2% male, 41.6% female, 4.2% no response; *M_*age*_* = 18.6 years, *SD* = 0.89; class standing: 64.3% Year 1, 22.7% Year 2, 7.1% Year 3, 1.7% Year 4, 4.2% no response).

### Procedure

As in Study 2, participants were randomly assigned to one of three conditions: Develop (*n* = 78), Fit (*n* = 81), and Well-Being (*n* = 79). They read an article corresponding to their condition and summarized its main message for a fellow student at the same school. The develop mindset and fit mindset articles used in this study were the same as those used in Study 2, and comparable on all characteristics other than their condition-relevant content. After reading the article, students completed a saying-is-believing exercise by writing a note sharing the message of the article to a fellow student from the same school – someone who shared a similar experience with the participant, instead of just a generic younger student as done in Study 2. This was done to motivate participants to want to help someone similar to themselves, and hence put more effort into internalizing and conveying the message of the articles. Students completed our multi-item develop and fit mindset scales, which served as our manipulation checks, at the end of the survey, after multiple filler questions to reduce demand effects.

## Results

### Message Content

Two independent raters, blind to hypotheses and conditions, coded the messages students wrote for content related to the develop mindset (*κ* = 0.83) and the fit mindset (*κ* = 0.86). Discrepancies were resolved through discussion. Participants in the Develop condition were much more likely to mention the develop mindset than those in the Fit condition (Develop condition: 87.0%; Fit condition: 0%), χ^2^(1) = 120.49, *p* < 0.001. Those in the Fit condition were much more likely to mention the fit mindset in their messages than the Develop condition (Develop condition: 7.8%; Fit condition: 92.4%), χ^2^(1) = 111.69, *p* < 0.001. In other words, students on average did read, understand, and accurately summarize the main message of their respective articles.

### Mindset Scales as Manipulation Checks

We averaged the items in the develop mindset and fit mindset scales separately (develop mindset scale: α = 0.81; fit mindset scale: α = 0.70). Participants in the Develop condition (*M* = 4.96, *SD* = 0.72) endorsed the develop mindset more strongly than those in the Fit condition (*M* = 4.44, *SD* = 0.88), *b* = 0.52 [0.26, 0.78], *t*(151) = 4.00, *p* < 0.001. Students’ endorsement of the fit mindset scale were higher in the Fit condition (*M* = 4.91, *SD* = 0.73) than the Develop condition (*M* = 4.64, *SD* = 0.81), *b* = 0.28 [0.03, 0.52], *t*(152) = 2.22, *p* = 0.031. These results suggest that the develop mindset and fit mindset articles were effective at changing students’ develop mindset and fit mindset, respectively, relative to the other condition.

## Part 2: One-Year Follow-Up

### Participants

One year later, we invited all participants from Part 1 of the study to participate in a survey in exchange for a raffle ticket toward prize money. We excluded from our analysis one participant who took our Part 2 survey who had completed our Part 1 survey twice. A total of 112 of the original participants participated and were used in our analyses in Part 2 (out of those who responded to the demographic questions, 57.1% female; *M_*age*_* = 19.7 years, *SD_*age*_* = 0.94; class standing: 2.0% Year 1, 59.2% Year 2, 26.5% Year 3, 9.2% Year 4, 3.1% other class standings). These participants did not differ in either their develop or fit mindsets from those who did not take this follow-up, *p*s > 0.230.

### Procedure

To report their intentions to use various cultivation strategies, students read the same vignettes as in Study 2 and made two ratings per strategy: how likely they would be to use the strategy when their passion is low, and how effective they thought the strategy would be at increasing passion. Both items were highly correlated across cultivation strategies (*r*s ranged from 0.67 to 0.86, *p*s < 0.001), so we averaged the two ratings per strategy, and summed them across all five cultivation strategies to form a “cultivation strategy-use intentions” composite score per student.

### Results

In total, 1 year after the mindset induction, students originally assigned at random to the Develop condition reported on average greater intentions to use cultivation strategies when their passion was low (*M* = 27.61, *SD* = 3.75), compared to those in the Fit condition—a marginally significant effect (*M* = 25.57, *SD* = 4.74), *b* = 2.04, [0.02, 4.11], *t*(68) = 1.97, *p* = 0.053, Cohen’s *d* = 0.48. Although our sample size after 1 year was small (due to natural attrition over time and practical constraints in our Part 1 recruitment), we were able to detect this medium effect size 1 year after the mindset manipulation. This finding replicated the short-term effect found in Study 2, and moreover, it gives us a practically important takeaway: On average, priming a develop mindset, even briefly, can significantly change people’s intentions to use effective strategies to cultivate their passion when it is low, even up to a year later. Although the effect size could differ by individual instilling this develop mindset (relative to fit mindset of passion) seems to have both short- and long-term effects on the average student’s cultivation strategy-use intentions.

To examine which strategies might have driven the effect, we additionally present exploratory results broken down by individual cultivation strategy in Table 4. In particular, students in the Develop condition reported greater average intentions to relate the subject to their personal goals, *b* = 0.73, [0.18, 1.27], *SE* = 0.27, *t*(68) = 2.69, *p* = 0.009, relative to the Fit condition. This particularly enduring effect is especially notable because it is consistent with decades of scientific research showing that personal relevance, or utility value (Eccles, 2009), increases students’ interest toward – and even academic grades in – a subject (e.g., Hulleman and Harackiewicz, 2009; Hulleman et al., 2010).

**TABLE 4:** Study 3 group means (Standard Deviations) and regression contrasts comparing participants’ cultivation strategy-use intentions between the Develop and Ft conditions 1 year later.

Strategy	Develop condition	Fit condition	Planned contrast (*b* and 95% CI)
(a) Personal relevance	5.77 (1.05)	5.04 (1.19)	0.73 [0.19, 1.27]**
(b) Societal relevance	5.45 (0.94)	5.05 (1.19)	0.40 [−0.12, 0.92]
(c) Build familiarity	5.17 (1.32)	4.92 (1.23)	0.25 [−0.36, 0.86]
(d) Practical experience	5.36 (1.20)	5.20 (1.48)	0.16 [−0.49, 0.81]
(e) Teachers or environments	5.86 (1.06)	5.36 (1.38)	0.50 [−0.09, 1.10]

Note: Unstandardized beta coefficients are presented with their 95% confidence intervals in square brackets. **p < 0.01.

## Discussion

Study 3 used a year-long, two-wave study to address the common question that is often asked about the duration of intervention effects in the context of priming mindsets about passion. Our results replicated, and extended, Study 2’s causal findings to long-term self-regulation. We showed that our brief, one-session develop-mindset induction was effective at changing students’ develop mindset on average – both in the short-term and 1 year later. Importantly, compared to the Fit condition, the effect of our develop mindset induction increased students’ intentions to use cultivation strategies, even up to a year later. Granted, due to the small sample size left after natural attrition 1 year later, this study was possibly underpowered. Future studies that do not have the constraints we faced should replicate these results with a larger sample.

## General Discussion

We started with a question: What can students do to ignite their passion toward their subjects when it burns low? Beyond relying on factors such as their external environment, prior task experience, or personality traits, those who hold a develop (vs. fit) mindset report using and intending to use cultivation strategies to grow their passion. Across three studies, a develop mindset correlationally and causally predicted greater intentions to use cultivation strategies (such as relating the subject to personal goals or seeking out inspiring teachers) to up-regulate one’s passion when it is low. Relative to priming a fit mindset, priming a develop mindset influenced the average student’s intentions to use these strategies in-the-moment, and also long-term 1 year later. Exploratory analyses showed that a develop mindset (relative to a fit mindset) oriented students toward focusing on the personal relevance of the subject as an especially enduring strategy. Changing students’ mindsets about passion could be a potentially fruitful way of increasing the likelihood that people would apply effective strategies to manage their passion when it burns low.

The present research contributes to existing work on mindsets about passion by illuminating a mechanism through which the develop mindset works to increase passion. Prior to this, findings showed that people with a develop mindset tend to believe that their passion would increase over time toward a line of work that they do not presently enjoy (Chen et al., 2015). Here, we extended those findings to show *how*: people with a develop mindset are oriented toward using more active, deliberate cultivation strategies (such as focusing on the personal relevance of the activity, gaining practical experience, or seeking out inspirational mentors) to increase their passion. More broadly, this connects research on mindsets with the literature on self-regulatory strategies in a novel domain, highlighting how mindsets can act through the selection of effective self-regulatory strategies to predict well-being (beyond learning and performance outcomes, on which prior mindset research has focused).

Our findings also extend the generalizability of research on mindsets about passion beyond working adults (Chen et al., 2015), to an earlier developmental time point that matters for how young adults choose their career paths (e.g., Daymont and Andrisani, 1984; Robst, 2007). We found evidence that college students do hold these develop and fit mindsets, and that these mindsets have implications for how they respond to motivational challenges as they pursue their college majors. Future intervention research may target people’s mindsets and regulatory strategies when they are still in school, when these attributes may be easier to change before they enter the workforce where there could be heavier consequences for their choices and behaviors.

Because we focused specifically on how students actively and purposefully up-regulate their passion, a develop mindset was especially relevant. This is consistent with the literature on growth mindsets, which has shown that holding a growth mindset (but not a fixed mindset) about a personal attribute is beneficial for increasing motivation, self-regulation, and even achievement (e.g., Hong et al., 1999; Burnette et al., 2013; Yeager et al., 2014b; O’Keefe et al., 2018). These results are not at all meant to imply that a fit mindset is useless. On the contrary, we believe that a fit mindset has regulatory value under the right circumstances (Chen et al., 2015; Chen and Ellsworth, 2019). To complement this work, future research could focus on better understanding the pros and cons of a fit mindset. For example, people with a fit mindset may be more selective about their engagements, and perhaps also more willing to leave a less-than-optimal situation earlier for a better option, compared to those with a develop mindset who may stick out inconveniences and troubles longer – for better or for worse.

Because we were interested in the strategies people take to actively up-regulate passion, this research focused on generally autonomous (rather than controlled) forms of motivation (see Vallerand, 2017). Such volitional and intrinsically motivated behavior, in turn, is associated with greater well-being (Vallerand, 2010, 2017; Vallerand and Houlfort, 2019). Importantly, this work shows that these specific up-regulatory strategies seem to be more strongly related to a develop (instead of a fit) mindset. Hence, the process by which a develop mindset works is through such autonomous behavior to actively grow passion. We cannot rule out that a fit mindset may be related to other autonomous strategies for seeking out passion, which future research should examine.

In related work on students’ mindsets about interest, O’Keefe et al. (2018) found that people tend to view interests as either dispositional and relatively unchanging (a “fixed” theory) or as malleable (a “growth” theory). In their research, when pursuing a new interest became difficult, college students with a growth theory maintained their interest, whereas those with a fixed theory no longer found the topic as interesting (O’Keefe et al., 2018, Study 5). Although this research did not investigate what specific strategies students might have employed to sustain their interest, their results on interest and ours on passion appear to converge on the same psychological process: a develop mindset of passion (and similarly a growth theory of interest) should predict the use of cultivation strategies that actively maintain passion (or interest) when it wanes. Given the connection between interest and passion, we believe that theorizing in both of these areas can be complementary in building our scientific understanding of how people find, cultivate, and ultimately achieve their passions.

Although our develop mindset article was successful at increasing students’ endorsements of the develop mindset across Studies 2 and 3, our fit mindset article was only effective at increasing students’ endorsements of the fit mindset in Study 3. It is worth considering that many of these effects of priming a develop mindset might be, in part, due to introducing the less well-known develop mindset message to people in a culture where the fit mindset is already prevalent and salient (see Chen et al., 2015). Future research could explore the questions: Would the fit mindset message show larger effects in cultures where the fit mindset is not the norm? Are there other, better ways to instill a fit mindset, even in situations where the fit mindset is already the default? What would the effects of these mindsets look like in different cultures?

Our findings suggest that priming a develop mindset with a persuasive article can have effects on people’s intentions to use certain cultivation strategies, some of which even last up to a year later. Nevertheless, some of these effects do seem to weaken over time, and we do not know for certain if students’ self-reported intentions translated into actual behavior. Future research could build upon the brief article and writing exercise that we used in our studies to design more extensive interventions that are even more effective at instilling a develop mindset, and also systematically measure changes in actual strategy-use behavior if possible (e.g., Oyserman et al., 2002; Crum et al., 2013).

## Conclusion

The value of experiencing passion and prescriptions to attain it has soared in the past decade. Our studies highlight that, in addition to people’s less controllable social environments and personality traits, people’s mindsets about passion matter too. The encouraging news is that a develop mindset of passion seems to be malleable, and priming it, even briefly, can bring about some long-term self-regulatory benefits. Studying people’s mindsets about passion offers a promising avenue for helping people take control of keeping the flames of passion alive in their lives.

## Data Availability Statement

Data and code are available at https://osf.io/rusfk/?view_only=e6478ccbf04d49599769895552a40fdb.

## Ethics Statement

The studies involving human participants were reviewed and approved by the University of Michigan and Stanford University. The Ethics Committee waived the requirement of written informed consent for participation.

## Author Contributions

PC, YL, and JY designed the studies. PC and YL collected the data. PC, YL, and DP performed the data analyses and contributed to writing the manuscript. PO’K and JY provided critical feedback during manuscript revision. All authors contributed to the article and approved the submitted version.

## Conflict of Interest

The authors declare that the research was conducted in the absence of any commercial or financial relationships that could be construed as a potential conflict of interest.
