# Supplementary material for: Fanning the Flames of Passion: A Develop Mindset Predicts Strategy-Use Intentions to Cultivate Passion
Source: Front Psychol. 2021 Jul 14;12:634903. doi: 10.3389/fpsyg.2021.634903 (PMC8375551; doi:10.3389/fpsyg.2021.634903)
Supplement: Supplementary file 1 [file Data_Sheet_1.pdf]

Supplementary Online Materials (SOM) for "Fanning the Flames of Passion: A Develop Mindset Predicts Strategy-Use Intentions to Cultivate Passion"

Table S1

Study 2 Demographic Breakdown of Samples From A Large Public Midwestern University and a Community College on the West Coast

Variable	Public Midwestern University		Community College	
	***N* (%)**	***M* (*SD*)**	***N* (%)**	***M* (*SD*)**
Age				
		19.0 (0.9)		24.6 (7.4)
Gender				
Female	22 (44.0%)		73 (72.3%)	
Male	28 (56.0%)		26 (25.7%)	
Other			2 (2.0%)	
Ethnicity				
African American/Black	4 (8.0%)		4 (4.0%)	
Asian/Asian American/Pacific Islander	3 (6.0%)		31 (30.7%)	
Caucasian/White	39 (78.0%)		31 (30.7%)	
Latino/Hispanic	3 (6.0%)		23 (22.8%)	
Middle Eastern	1 (2.0%)		3 (3.0%)	
Multiracial/Biracial/Mixed Race			6 (5.9%)	
Others			3 (3.0%)	
Class Standing				
Freshmen	36 (72.0%)		16 (15.8%)	
Sophomore	9 (18.0%)		42 (41.6%)	
Junior	3 (6.0%)		14 (13.9%)	
Senior	2 (4.0%)		13 (12.9%)	
Graduate student			4 (4.0%)	
Other			12 (11.9%)	

Note. One participant did not provide any demographic responses, and is missing from this breakdown (N = 151).

Table S2

Study 2 *Exploratory Analyses (Not For Hypothesis-Testing): Group Means (Standard Deviations) and Regression Contrasts Comparing Participants' Cultivation Strategy-Use Intentions Between the Develop and Well-Being conditions (D vs. WB contrast), and Between the Fit and Well-Being conditions (F vs. WB contrast)*

**Strategy**	**Develop condition**	**Fit condition**	**Well-Being condition**	**D vs. WB contrast (*b* and 95% CI)**	**F vs. WB contrast (*b* and 95% CI)**
**(a) Personal relevance**	5.35 (1.02)	4.74 (1.44)	4.96 (1.50)	0.40 [-0.13, 0.93]	-0.22 [-0.75, 0.32]
**(b) Societal relevance**	5.32 (1.18)	5.09 (1.13)	4.88 (1.22)	0.44 [-0.03, 0.91]	0.20 [-0.28, 0.67]
**(c) Build familiarity**	4.93 (1.37)	4.01 (1.58)	4.59 (1.27)	0.34 [-0.23, 0.91]	-0.60 [-1.18, -0.02]*
**(d) Practical experience**	5.72 (0.98)	5.12 (1.41)	4.92 (1.38)	0.80 [0.30, 1.30]**	0.17 [-0.34, 0.68]
**(e) Teachers or environments**	5.81 (0.87)	5.35 (1.16)	5.47 (1.14)	0.33 [-0.09, 0.76]	-0.12 [-0.54, 0.31]

Note. The critical comparison in our paper was the comparison between Develop and Fit mindset conditions. Here, we present exploratory analyses testing their differences with a third condition that primes only well-being and not either of the mindsets of passion. The Well-Being condition was used as the reference group for both the D vs. WB contrast and the F vs. WB contrast (i.e., positive b coefficients denote that the Develop/Fit condition scored higher). Both contrasts were included in each regression model. For each contrast, unstandardized beta coefficients are presented with their 95% confidence intervals in square brackets. * $p < .05$; ** $p < .01$.

Table S3

Study 3 Exploratory Analyses (Not For Hypothesis-Testing): Group Means (Standard Deviations) and Regression Contrasts Comparing Participants' Cultivation Strategy-Use Intentions Between the Develop and Well-being conditions (D vs. WB contrast), and Between the Fit and Well-Being conditions (F vs. WB contrast)

**Strategy**	**Develop condition**	**Fit condition**	**Well-Being condition**	**D vs. WB contrast (*b* and 95% CI)**	**F vs. WB contrast (*b* and 95% CI)**
**(a) Personal relevance**	5.77 (1.05)	5.04 (1.19)	5.72 (1.18)	0.04, [-0.54, 0.62]	-0.69, [-1.24, -0.13]*
**(b) Societal relevance**	5.45 (0.94)	5.05 (1.19)	5.48 (1.11)	-0.03, [-0.59, 0.53]	-0.43, [-0.96, 0.10]
**(c) Build familiarity**	5.17 (1.32)	4.92 (1.23)	5.02 (1.42)	0.16, [-0.52, 0.82]	-0.10, [-0.74, 0.55]
**(d) Practical experience**	5.36 (1.20)	5.20 (1.48)	5.74 (1.15)	-0.38, [-1.05, 0.28]	-0.54, [-1.18, 0.09]
**(e) Teachers or environments**	5.86 (1.06)	5.36 (1.38)	6.07 (1.01)	-0.21, [-0.81, 0.39]	-0.71, [-1.29, -0.14]*

Note. The critical comparison in our paper was the comparison between Develop and Fit mindset conditions. Here, we present exploratory analyses testing their differences with a third condition that primes only well-being and not either of the mindsets of passion. The Well-Being condition was used as the reference group for both the D vs. WB contrast and the F vs. WB contrast (i.e., positive *b* coefficients denote that the Develop/Fit condition scored higher). Both contrasts were included in each regression model. For each contrast, unstandardized beta coefficients are presented with their 95% confidence intervals in square brackets. * $p < .05$.

SOM Appendix A

Study 1 Examples of Students' Open-ended Responses

Some examples of students' responses that captured building familiarity with the subject matter included: "The more I learned about it, the more I realized how much I enjoyed it" and "Learning more about feminism has only made me more intrigued and dedicated to the topic." Examples of students' responses about the motivational impact of choosing inspiring teachers or conducive learning environments included: "I have some great friends that are very passionate about the topic and they inspire me to learn more about it," "The professors and the community within the major made it easy to learn from one another while pushing each other to learn", and "meeting awesome professors." Examples of students' responses that emphasized the societal relevance of the subject included: "I knew I was interested in the topic and the more I learned, the more I appreciated the subject in terms of real-world impact" and "The deeper my classes delved into economics, the more interesting it became and the more I was able to apply it outside the classroom. Especially when I started getting into business news, I get excited when I know what they're talking about." Examples of students' responses categorized as recognizing the relevance of the subject to personal goals included: "focused on aspects of the subject that interest me and are relevant to my goals (global health)" and "I was able to see how the material I was using could be applied to my current job and my future professional aspirations". Examples of students' responses categorized as gaining practical experience in applying the subject were: "The hands on experiences including research and internship" and "my experience at my internship helped to grow my passion in mechanical engineering." Examples of students' responses categorized as identifying parts of the subject that they naturally liked were: "I get to pick the psychology courses that I find more interesting instead of taking courses that are

required" and "finding which fields of biology I enjoy," Examples of students' responses categorized as focusing on performing well in the subject included: "Performing well," "Success in early classes," and "It really difficult but I can do it well. And I did well and I feel great."

SOM Appendix B

Study 1 Results for the Relation Between a Develop Mindset and Students' Reported Use of Each Individual Cultivation Strategy

Examining individual strategies, we found that the more students endorsed a develop mindset, the more likely they were to describe actively recognizing the societal relevance of the subject (b = 0.63, SE = 0.27, Wald(1) = 5.55, p = .018, odds ratio = 1.87, 95% CI for odds ratio: [1.11, 3.15]), and gaining practical experience related to the subject (b = 1.09, SE = 0.36, Wald(1) = 9.31, p = .002, odds ratio = 2.97, [1.48, 5.97]). Although only marginally statistically significant, the develop mindset was also positively related to other cultivation-oriented strategies in practically meaningful ways: Stronger endorsement of a develop mindset was associated with actively building greater familiarity with the subject (b = 0.28, SE = 0.16, Wald(1) = 3.15, p = .076, odds ratio = 1.32, [0.97, 1.79]), identifying personal relevance in the subject (b = 0.52, SE = 0.31, Wald(1) = 2.88, p = .090, odds ratio = 1.68, [0.92, 3.07]), and being inspired by the positive influence of their teachers or educational environments (b = 0.36, SE = 0.22, Wald(1) = 2.79, p = .095, odds ratio = 1.44, [0.94, 2.20]). To illustrate the effect size: with every unit increase in their endorsements of a develop mindset, students were on average 1.75 times more likely to mention identifying personal relevance in the subject as a strategy they had used to up-regulate their passion. As expected, endorsements of the develop mindset were neither related to focusing solely on parts of the subject that they naturally liked, nor to high performance in the subject, $ps > .510$.

Appendix C
Develop Mindset Article

Experiencing Passion: Is it love at first sight or a cultivated process?

University of Michigan researchers find that students' and working professionals' passion for their fields develops gradually over time.

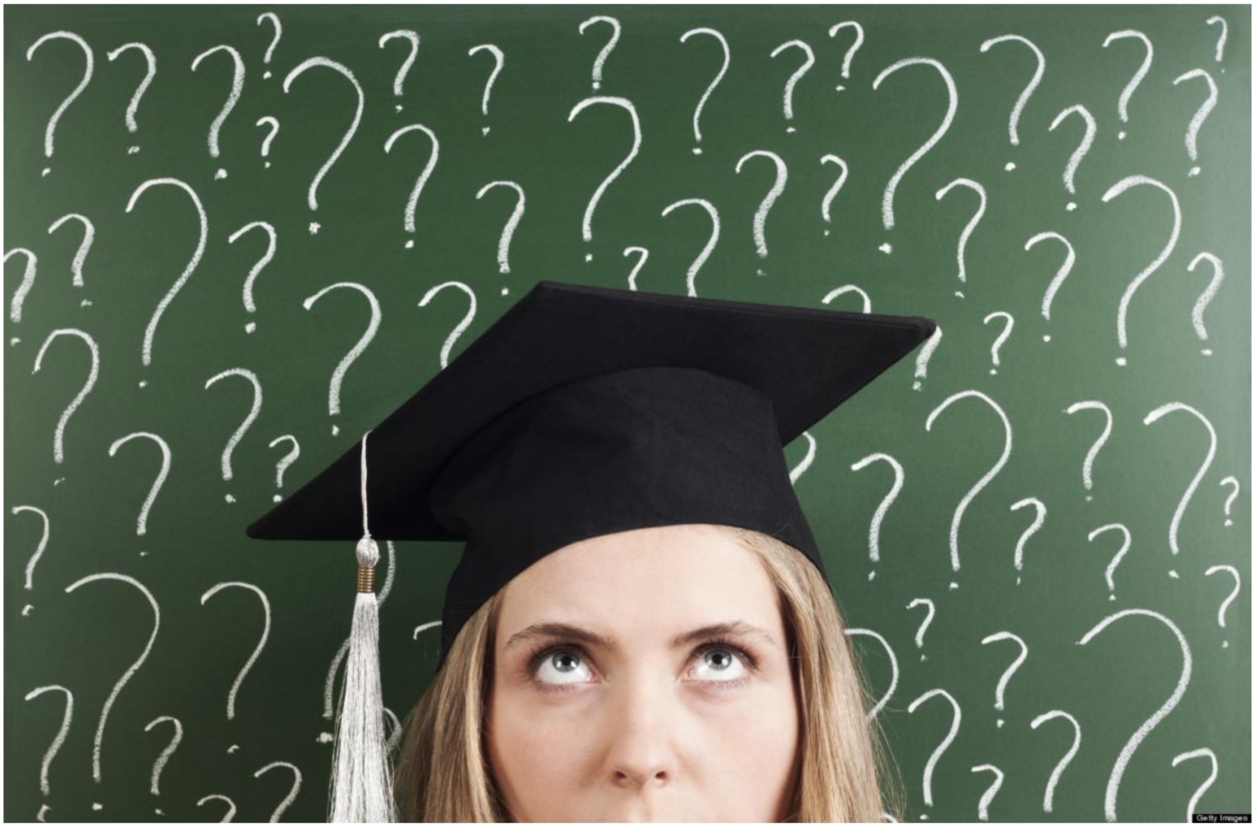

Carmen Lin | May 23, 2016

To students and working professionals who have yet to find their passion: Take heart. Psychologists have found that feeling passionate about a line of work isn't usually a love-at-first-sight experience. For most, passion actually develops over time.

In a recent ten-year study published in *Psychological Science,* a team of scientists from the University of Michigan led by Professor Richard Clarkson surveyed hundreds of working adults

from multiple Fortune 500 companies. By the end of the ten-year period, the researchers found that people who strongly believed that “their passion towards their line of work was something that *grew gradually over time*" reported being *43% more passionate and motivated* in their professions than those who had initially disagreed with the statement.

This study confirms findings in a series of papers published over the past two decades on vocational well-being. Clarkson emphasized that, “When people don’t start off enthusiastic about their profession, it is in no way a deal breaker.” Clarkson explained, “A large body of research generated during the past twenty years has demonstrated that the more commitment and effort people put into their work, the more competent they become at it. When people are good at something, they tend to like it more and recognize how relevant it can be to the world. Over time, experience and dedication accumulate into strong passion and conviction towards the profession.”

Many educators and academic advisors at various academic institutions are already trying to incorporate these research findings into what they tell their students. For example, these advisors tell students in advance not to expect love-at-first-sight experience in every class they take, but to understand that passion for a subject (just like expertise) takes time and effort to grow.

“The more commitment and effort people put into their work, the more competent and interested they become in it… Over time, experience and dedication accumulate into strong passion and conviction.”

Take Sylvia Walton, a Chemical Engineer at a pharmaceutical company in Buffalo, New York, for example. “Learning to truly love Chemistry the way I do now definitely wasn’t an overnight process,” she laughed. “Before my first college Chemistry class, I had heard all these stories of people getting bad grades and hating the class. It definitely made me feel a lot of pressure. At the beginning, I, too, questioned whether I could ever enjoy or do well in the subject at all, just like everybody else.”

So how did her feelings change? “It took time and experience. I really worked hard to learn, I joined a research lab, I spoke with different faculty members and anyone I knew who happened to be a Chemist, and I did a lot of reading. The more I understood about Chemistry and the

more time I spent with people who were deeply involved in it, the more passionate I came to feel about the subject too. I remember that, by the end of my first summer internship at Eli Lilly, I felt like I knew a lot more and I appreciated just how much one can do with a background in Chemistry," she reflected.

Aaron Gomez, a top graduate in Economics from the University of Michigan in 2014, had an experience similar to Sylvia's. He did not start out planning to become an Economics major in his first year, let alone think that he would want a job in the industry after graduation.

"To be honest, I didn't particularly like my Econ classes as a freshman. I didn't find the material engaging and I seriously considered changing majors. But after taking a few more classes in it and realizing how it is relevant to problems in the world, I started to appreciate it more and more." Aaron said that he especially noticed this change in his feelings about Econ towards his junior year when he was already taking more advanced courses. "I guess I learned to enjoy the feeling of solving challenging real-world problems. Then after some time, I realized that I really wanted to do this as a career."

In another longitudinal study of college students, Harvard Professor Henry Yates and his team interviewed and followed 371 undergraduates throughout their college careers and five years into their post-graduate jobs. "We found that the majority of students did not necessarily start out zealous towards the very subjects that they chose to major in, or even the professions they chose later on," Yates shared. "Sure, some students were lucky and loved their subject right from the start, but these are a minority of cases. The data overwhelmingly show that for most people, becoming passionate about something requires gaining more experience in it and developing a mature understanding of how it is relevant to the world."

In short, passion towards any subject or line of work develops through a gradual process of learning about it, building familiarity with it, and gaining experience in it. This is heartwarming news for many of us who are struggling to get through the semester of uninteresting lectures or uncertain about our career paths. The science shows that we can learn to love our subjects or lines of work over time. As with many other things in life, it takes dedication and patience.

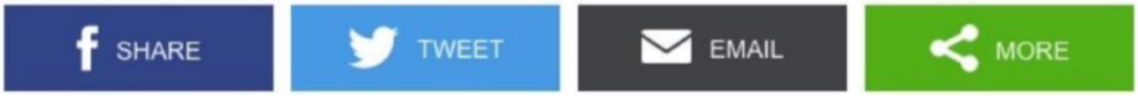

Fit Mindset Article

Finding Your Passion: Is it love at first sight or a cultivated process?

University of Michigan researchers find that students' and working professionals' passion for their fields is found through a fit with the right line of work.

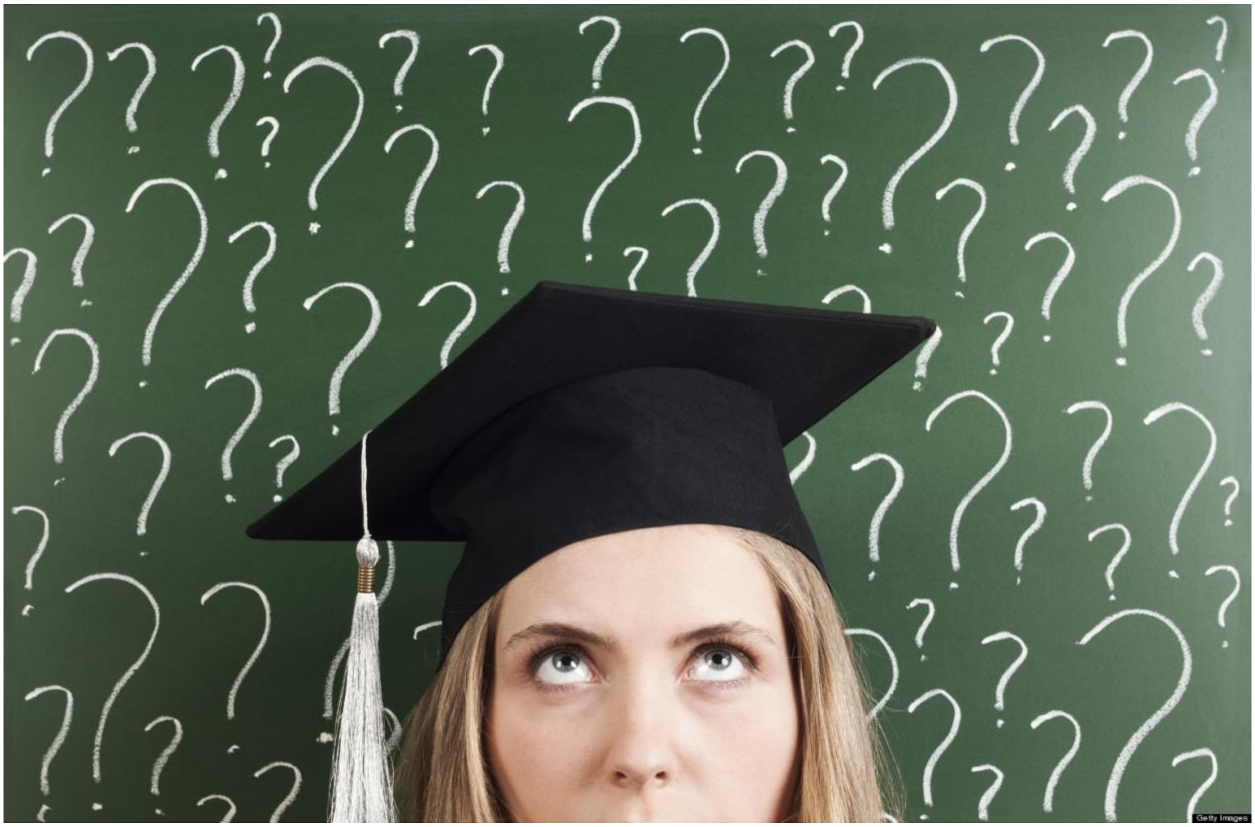

Carmen Lin | May 23, 2016

Psychological research offers us new findings and heartening advice for people who have yet to find their passion. The bottom line: Passion comes about from finding a line of work that is the right fit for you.

In a recent ten-year study published in *Psychological Science,* a team of psychologists from the University of Michigan led by Professor Richard Clarkson surveyed hundreds of working adults from multiple Fortune 500 companies. By the end of the ten-year period, the researchers found that people who strongly believed that their career was "*highly compatible* with the kind of

person they were" reported being *43% more passionate and motivated* in their professions than those who had initially disagreed with the statement.

The study confirms findings in a series of papers published over the past two decades on vocational well-being. Clarkson emphasized that, "When people find the line of work that really aligns with who they are as a person, they are naturally motivated to dedicate a lot more time to their work, and they are more engaged in it. They themselves want to do the work and do it well." Clarkson explained, "People naturally want to do what 'feels right' to them. And for good reason--there is a large body of scientific evidence showing that people's first impressions tend to be surprisingly accurate."

Many educators and academic advisors at various academic institutions are already trying to incorporate these research findings into what they tell their students. For example, these advisors tell students in advance to think deeply about what their personal interests and strengths are, and what careers they might consider, and then to explore which subjects suit them best.

"When people find the line of work that really aligns with who they are as a person, they are naturally motivated to dedicate a lot more time to their work, and they are more engaged in it."

Take Sylvia Walton, a Chemical Engineer at a pharmaceutical company in Buffalo, New York, for example. She realized that she had a passion for the subject during her Introductory Chemistry classes as an undergraduate student. "I remember sitting in my first Chem class my freshman year and just being fascinated by it. I was immediately intrigued by the concepts covered in class. I loved how much my classes dealt with questions that I personally thought about a lot."

How did her motivation sustain itself from those first few classes into her career today? "I was just naturally passionate about the subject. I knew early on that this field was what I wanted to go into because I really enjoyed learning about it through my readings and class lectures. The sense of fit was really apparent to me when I took my first few classes in Chem. I definitely did not feel as excited about my other subjects. I guess that when you're in a major that really fits you well, you just naturally feel passionate about it and constantly want to learn more and get better at it," she shared.

Aaron Gomez, a top graduate in Economics from the University of Michigan in 2014, had an experience similar to Sylvia's. He did not start out knowing what major he wanted to pursue in his first year, let alone the career that would follow after graduation.

"I started out thinking I was going to study Biology, but I just could never get myself into the material. After taking my first Econ class, it just clicked. I was just so interested in the topics that we covered and I kept seeing how relevant it was to problems in the real world." Aaron said that he naturally thought about things in the same way that Economists reason about issues. "I was personally drawn to solving challenging real-world problems with the kind of logic that I learned through Economics. It's a very elegant and rational way of thinking about how people, organizations, and markets work. I guess it didn't take me long to realize that I really wanted to do this as a career."

In another longitudinal study of college students, Harvard Professor Henry Yates and his team interviewed and followed 371 undergraduates throughout their entire college careers into their post-graduation jobs. "We found that, by far, people who experienced the highest levels of passion towards their majors and their professions were those who knew that they were naturally passionate about the field from the very beginning," Yates shared. "Not everyone found their fit right away, but it was strikingly evident that once they did find the right fit with their interests, people felt very passionate about what they were doing--and this passion sustained itself for a long time."

In short, it is possible to find the right line of work (and college major) for you personally. Many working students and professionals have found their passions in careers that are compatible with their interests and personalities. This is heartwarming news for many of us who are struggling to get through the semester of uninteresting lectures or uncertain about our career paths. The science shows that we can find our passion--it's mostly about listening to our intuitions and looking out for the right fit.

SHARE TWEET EMAIL MORE
